# Different drying methods of *Pistacia Atlantica* seeds: Impact on drying kinetics and selected quality properties

**DOI:** 10.1002/fsn3.1582

**Published:** 2020-05-20

**Authors:** Negar Yarahmadi, Mohammad Hojjatoleslamy, Leila Sedaghat Boroujeni

**Affiliations:** ^1^ Department of Food Science and Technolog, Shahrekord Branch Islamic Azad University Shahrekord Iran

**Keywords:** drying, mathematical modeling, microwave, moisture, *Pistacia atlantica*

## Abstract

The effect of different drying procedures on the quality characteristics of *Pistacia atlantica* subsp. *kurdica* is addressed in this work. Using five different drying methods include microwave, oven (40 and 60°C), sun, and shade, *P. atlantica* were dried. The variations in moisture content, drying rate, major components of essential oil, and texture property were assessed at the start and at the end points of the drying process. Comparison of the drying methods indicated that microwave drying to be most effective in lowering moisture content, while the shade drying had the lowest rate among methods. In the case of microwave, the dried seeds had highest brittleness, while the highest score for the penetration force was observed in oven 40°C. Regarding major elements of the essential oil (α‐pinene, α‐terpinen‐4‐ol, myrcene, β‐ocimene, β‐caryophyllene, and limonene), there were no significant differences between the five drying techniques, nor compared to the fresh sample. To model the drying process, six thin‐layer drying kinetic models were chosen. It was found that the Midilli–Kucuk model was the most suitable for explaining the drying curve of oven 40 and 60°C, microwave, and sun methods; Wang and Singh model was excellent to explain thin‐layer shade drying behavior of the *P. atlantica* seeds. In conclusion, in this study, an opportunity is represented to apply the most effective procedures to decrease the drying period and to achieve a product with appropriate safety and quality features.

## Graphical abstract

The Midilli–Kucuk model was found to be the most suitable for describing drying curve of oven 40 and 60°C, microwave, and sun methods; and Wang and Singh model was found to best explain thin‐layer shade drying behavior of the *P. atlantica* seeds. This study represents an opportunity to apply the most effective procedures to reduce the drying period.

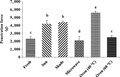

## 1 INTRODUCTION

Nowadays, there is a trend for dried vegetables and fruits and expected to continue over the following. Drying provides a tool preserving products in a stable and safe condition by reducing its moisture content and water activity, also enhances storage stability, minimizes packaging uirement, and facilitates transportation (Karunasena, Brown, Gu, & Senadeera, 2015; Larrosa, Cadaval, & Pinto, 2015; Law, Chen, & Mujumdar, 2014). Dried products have much longer shelf life and they can be stored for months or even years without appreciable loss of nutrients, compared to fresh products which can be only kept for a few days under appropriate conditions (Ortiz‐García‐Carrasco et al., 2015). The removal of moisture from the food materials inhibits the microbial spoilage, slows down the enzymatic reactions, and minimizes most of the deteriorative reactions mediated by the moisture (Aguilera, Chiralt, & Fito, 2003; Oms‐Oliu et al., 2010); all of these improve quality and shelf life of product. The different ways of drying are natural, mechanical, or thermal drying. Artificial drying methods are more often used which make possible fast and efficient removal of huge deals of moisture (Carvalho, 1994).

*Pistacia atlantica* subsp. *kurdica* (Anacardiaceae), namely “Bene” in Iran, have been distributed throughout Zagross Mountains in Iran (Farhoosh & Tavassoli‐Kafrani, 2011). Bene fruit has been utilized for the various nutritional and medicinal purposes in folk medicine (Pourreza, Shaw, & Zangeneh, 2008). After grinding and mixing with other ingredients, it is used by local people as food and the immature fruits are used for jam preparation. Oleoresin of Bene, known as saqez in Iran, is used to produce chewing gum (Hatamnia, Abbaspour, & Darvishzadeh, 2014). In traditional medicine, some extracts of aerial and underground parts have been utilized for treatment of various conditions, such as strengthens gums, deodorizes breath, and relieves upper abdominal discomfort and pain, and stomach diseases (Bozorgi et al., 2013). Previous studies on *P.* *atlantica* showed that it contains flavonoids (Tzakou, Bazos, & Yannitsaros, 2007), fatty acids and triglycerides (Farhoosh, Tavakoli, & Khodaparast, 2008; Yousfi, Nadjemi, Belal, Bombarda, & Gaydou, 2005; Yousfi, Nedjmi, Bellal, Ben‐Bertal, & Palla, 2002), oleoresin (Benhassaini, Bendeddouche, Mehdadi, & Romane, 2008; Delazar, Reid, & Sarker, 2004), and essential oils (Barrero et al.., 2005; Tzakou et al., 2007). Scientific researches as well disclosed therapeutic effects of Bene seeds. Phenolic compounds of different parts of Bene had antioxidant activity (Hatamnia et al., 2014). Bene hull is regarded as a rich source of potent natural anticancer agent (Rezaei, Fouladdel, Ghaffari, Amin, & Azizi, 2012). In addition, *P. atlantica* had a role for treatment of chronic inflammatory bowel disease (Gholami et al., 2016).

*Pistacia atlantica* is dried traditionally by sun drying (Kaveh, Amiri Chaijan, Ahmadi, & Amiri Parian, 2012). Recent studies have focused on the new drying methods for *Pistacia atlantica.* They affect the physicochemical properties such as color (Pourahmadiyan, Hojjatoleslamy, Ghasemi Pirbaloti, Rooshank, & Shariati, 2015), texture, amount of essential oil obtained from the plant, and the composition of the essential oil, and it seems there is a requirement for developing the appropriate drying methods to shorten the drying time and to increase the shelf life of *P. atlantica* seeds. This work aimed to assess the effects of sun, shade, microwave, and oven (40 and 60°C) as drying methods on moisture content, essential oil contents of *P. atlantica*, and its texture. Many mathematical models have been suggested to predict the behavior of various plant tissues during the drying operation, and the most common models have been selected to describe the behavior of *Pistachio atlantica.* Then, the experimental data were fitted to six mathematical models to achieve the most appropriate model for drying.

## 2 MATERIALS AND METHODS

### 2.1 Materials

Fresh seeds of *Pistacia atlantica* subsp. *kurdica* (50 kg) were bought from a local market in Kermanshah province, Iran, and kept at 4°C. All other chemicals and solvents used were obtained from Merck.

### 2.2 Drying processes

The seed samples were subdivided into six groups after cleaning, and one of the groups was kept fresh as control; drying experiments were carried out using five different methods: oven (40 and 60°C), sun, shade, and microwave.

Drying through the 40 and 60°C oven: The sample was divided into 10 parts (each of 150 g) covered with aluminum foils and placed in an oven at 40 or 60°C, monitored each 30 min until fixing the weight of the sample and obtaining the moisture content of 2% and less.

The sample was divided into ten parts (each of 150 g) and spread in a monolayer on mesh sieves under direct sunlight for 60 hr (Karathanos &Belessiotis, 1997). The sieves were put in a bird‐ and rodent‐proof structure, made up of wooden framework covered by a fine wire meshwork inhibiting the entry of these pests, while allowing free airflow at the same time. The mean relative humidity was 12%, and the mean ambient temperature over the drying period was 29°C. Every 6 hr, variations in seed weight were monitored until reaching a constant moisture. Then, the dried sample was packaged into an aluminum foil packet until following tests.

Drying through the shade: The sample was divided into ten parts (each of 150 g), placed in aluminum foils and then in a ventilated room with an ambient temperature of 24°C and 12% relative humidity. The seed weight changes were checked every 6 hr until obtaining a 2% moisture.

Drying through microwave: Seeds were placed in Pyrex trays, and moisture contents of samples dried over microwave were monitored regularly at one min intervals until achieving the constant 2% moisture. Then, the specimens were packaged hermetically in vacuum‐packed aluminum foil packets and kept in a room at temperature within 20–25°C for following tests.

### 2.3 Determination of moisture content

Utilizing a digital moisture meter (Multi Grain), moisture measurements were carried out for each method, for fresh sample and in a specified time intervals during the drying procedure until to reach a constant weight. The moisture content was determined based on the AOAC standard procedure (AOAC, 1996). The moisture content was calculated after drying, (utilizing the mean of the two replicates) on dry weight basis based on equation 1:

(1) $$\text{Moisture ratio}\left(\text{MR}\right)=\frac{M_{t}-M_{e}}{M_{0}-M_{e}},$$

where *M*_t_, *M*_0,_ and *M*_e_ express the moisture content at any time within drying; primary moisture content; and equilibrium moisture content (kg_water_/kg_dry matter_), respectively.

Drying curves were determined through adjusting the curve concerning the alteration in seed moisture content and the drying process length (Kiranoudis, Tsami, Maroulis, & Marinos‐Kouris, 1997; Pourahmadiyan et al., 2015).

### 2.4 Essential oil extraction and gas chromatography–Mass spectrometry analysis

One hundred grams of the dried samples was separately transferred to the flask of a Clevenger apparatus. Then, about 1,500 ml of distilled water was added to the contents of the flask, and after installing the device connections; the oil extraction operation was allowed to be done at a temperature of 90°C, for 6 hr.

On Agilent Technologies‐7890A GC system combined with Agilent Technologies‐5975C mass spectrometer (GC–MS), chromatographic analysis was performed to identify the compounds of the essential oil (Sedaghat Boroujeni & Hojjatoleslamy, 2018). The system was tailored with a HP‐5MS capillary column (30.0 m × 0.25 mm × 0.25 µm), and helium was utilized as carrier gas at a flow rate of 2 ml/min with 0.1 µl injection volume. The specimens were analyzed with the column held initially 60°C with 10 min hold time, incremented to 240°C with 4°C/min heating ramp. The injection was conducted in splitless manner. Detector and injector temperatures were 280 and 250°C, respectively, and the run time was 80 min. The mass scan altered within 35–450 m*/z* at 70 eV. Essential oil components were identified comparing their retention times with those of authentic standards, moreover, by comparing their retention indices with those existed in the literature (Adams, 2001).

### 2.5 Texture

Using a texture analyzer (CT3; Brookfield Engineering), the texture properties of the dried samples were assessed (Atashkar, Hojjatoleslamy, & Boroujeni, 2018). The seeds were fractured with a 2‐mm cylindrical probe at a test speed of 1 mm/s. For each sample obtained from different drying conditions, 10 measurements were performed.

### 2.6 Mathematical modeling

Table 1 represents six well‐known thin‐layer drying models tested for describing the drying process of *P. atlantica* seeds. The coefficient (*R* ^2^) and root mean square error (RMSE) were determined as two major criteria for choosing the proper Equation (Arumuganathan, Manikantan, Rai, Anandakumar, & Khare, 2009; Togrul & Pehlivan, 2003).

(2) $$R^{2}={\frac{\sum {\left({\text{MR}}_{\text{pre,i}}-{\text{MR}}_{\text{exp,avg}}\right)}^{2}}{\sum ({\text{MR}}_{\text{exp,i}}{\text{-MR}}_{\text{exp,avg}})}}^{2},$$

(3) $$\text{RMSE}={\left[1/N\sum {\left({\text{MR}}_{\text{pre,i}}{\text{-MR}}_{\text{exp,avg}}\right)}^{2}\right]}^{2},$$

where MR_exp,i_ represents the experimental moisture ratio; MR_pre,i_ is the predicted moisture ratio; and *N* shows the number of observation.

**Table 1:** Mathematical models applied to drying curves of *P. atlantica* samples

Model name	Equation
Newton	MR = exp (−*Kt*)
Page	MR = exp (−*Kt* ^n^)
Henderson**–**Pabis	MR = a exp (−*Kt*)
Logarithmic	MR = a exp (−*Kt*) + c
Wang and Singh	MR = 1 + at + bt^2^
Midilli–Kucuk	MR = a exp (−*Kt* ^n^) + bt

Note

MR, dimensionless moisture ratio; a, b, c, and *n*, empirical coefficients in thin‐layer drying kinetic models; k, drying rate constant (1/s).

### 2.7 Statistical analysis

Data were analyzed through analysis of variance (ANOVA) using SPSS V.19.0. Duncan test indicated significant differences between samples. The least significant difference (LSD) test determined the significance of differences between means at *p* < .05.

## 3 RESULTS AND DISCUSSION

### 3.1 Impacts of drying methods on moisture content of *P. atlantica* seeds

**Figure 1:**
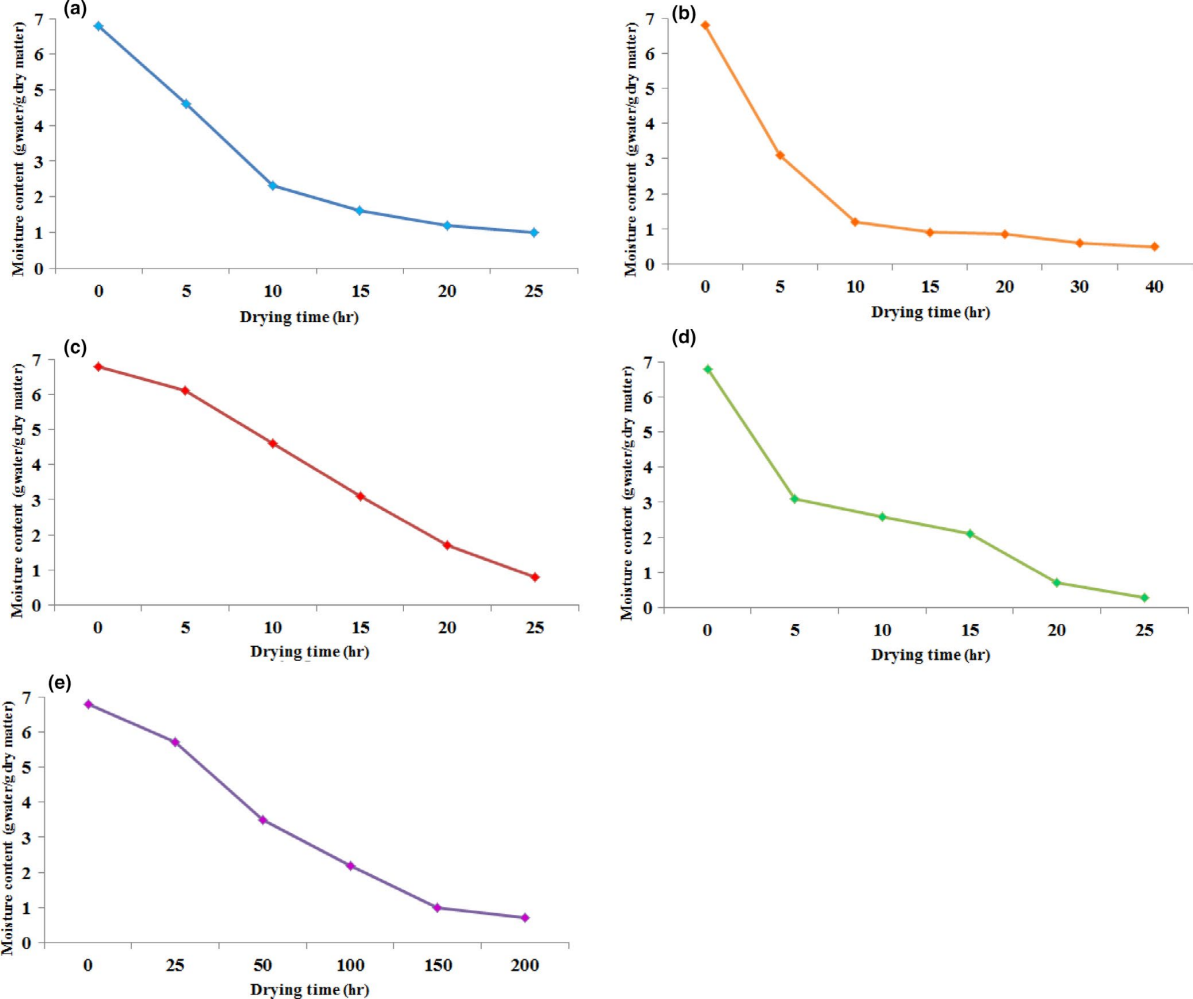
Changes in moisture content of *Pistacia atlantica* seeds under drying: (a) microwave, (b) sun, (c) oven (40°C), (d) oven (60°C), and (e) shade

Drying the food is performed at mild temperatures (between 40 and 70°C) to minimize bioactive compounds degradation (Fernandes & Rodrigues, 2007). Figure 1 shows the changes in the moisture content for the different methods. The baseline moisture content was 6.8 g water/g dry matter. As it is observed, the time of reduction in the moisture content is different in drying methods; however, in all methods, the drying was at a reducing ratio, growing with time (Figure 1). In microwave method, the moisture content decreased at high rate, and within 10 min, the moisture content reaches ~2% (Figure 1a). In sun drying (air temperature was ~29°C), moisture loss from the 5th to 20th hour was considerable, but reduced from the 20th to 40th hour (Figure 1b). In the oven method (for both temperatures), the moisture content reaches 2% during about 20 hr (Figure 1c,d). In fact, no significant impact on the drying rate was found by the increase in the temperature from 40 to 60°C for oven. Among the drying methods, the shade method had the lowest rate; in the shade drying, the time required to reduce seed moisture from 6.8% to ~2% was of 100 hr (Figure 1e).

**Figure 2:**
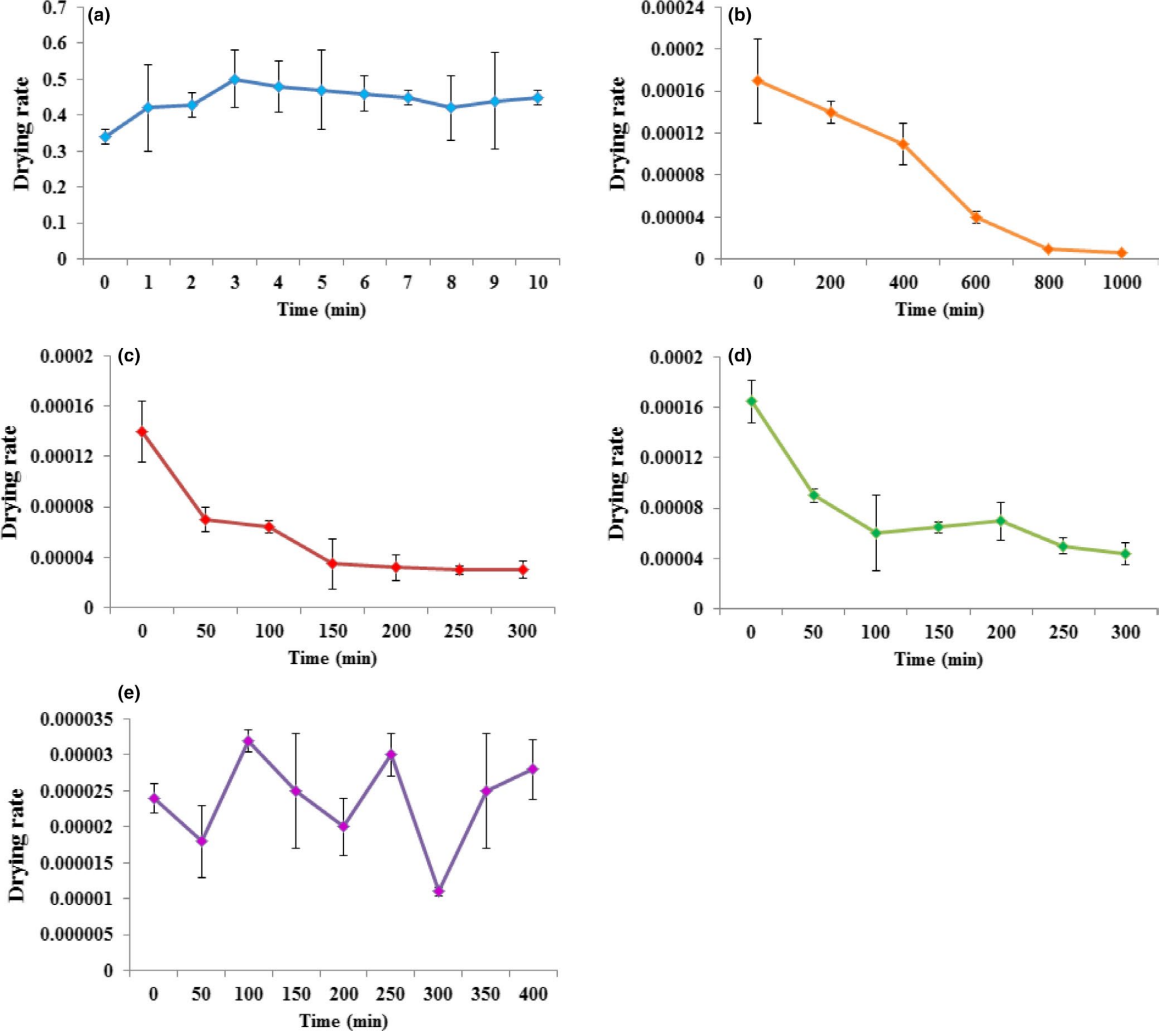
Drying rate of *Pistacia atlantica* seeds under drying: (a) microwave, (b) sun, (c) oven (40°C), (d) oven (60°C), and (e) shade

Figure 2 indicates the moisture ratios versus drying time for the five drying methods. Drying rate decreases in all methods, constantly with moisture content or drying time. It was illustrated by the analysis of the drying curves that the removal of moisture from seeds was slower in the microwave method and faster in the sun drying. For the microwave, drying rate not varied considerably throughout drying process (Figure 2a). Microwave drying can profit with its volumetric heating and reduced processing time (Figiel, 2010). During the initial phase of the drying, the moisture content of the material was high which led to a higher absorption of microwave power and higher drying rates as a result of the higher moisture diffusion. Continuing the drying, the moisture loss in the product led a reduction in the absorption of microwave power and caused a reduction in the drying rate. In the studies on carrot slices and parsley, similar results were reported (Soysal, 2004; Zarein, Banakar, & Khafajeh, 2013). Seeds dried through the sun method lost moisture more quickly compared to the other methods (Figure 2b). As observed in oven drying (40 and 60°C), low drying speed of *P. atlantica* seeds led to slow desorption and determined a long drying time (Figure 2c,d). The shade drying is inadequate for the reason that causes longer drying times, as found in the current study (Figure 2e). The slowness of the drying process in the shade condition is among the disadvantages of this method. If drying is too slow, diverse problems are possible such as the growth and proliferation of microorganisms, reduction in seed quality, and the nutritional compound loss (Raouzeos & Saravacos, 1986). In addition to the method, some other elements influence these differences, such as drying temperature, drying exposure times, humidity in the site, drying airflow, and the speed of transferring the moisture from the inner seed structures to its surface.

Numerous drying methods were suggested for other fruits and seeds. Convective air‐drying in an oven was investigated in fruits such as peaches, plums, grapes, and cherries as well as in vegetables such as celery, corn, carrot, tomato, and garlic (Krokida, Karathanos, Maroulis, & Marinos‐Kouris, 2003). In a study for comparing the efficiency of sun, shade, and silica gel drying in terms of rice seed *Oryza glaberrima* and *O. sativa,* sun drying most significantly reduced moisture content compared with others (Somado et al., 2006). For drying of thin‐layer carrot slices, microwave method significantly affected the drying time and drying rate (Soysal, 2004).

### 3.2 Effects of drying on major compounds of the essential oil of the *Pistacia atlantica* seeds

Extract of *P. atlantica* seeds yielded: 0.02%–0.12% (v/w) (Table 2) and was found as paleyellow to light‐brown color oils with aromatic‐spicy odor. The composition of essential oils from fresh specimens and samples dried using different methods was analyzed by GC–MS. Thirty compounds of the essential oils were recognized, and percentage range between 0.03% and 46.04% (not published in this work). Some studies exist on the chemical composition of *P. atlantica* from different origins in which α‐pinene, α‐terpinen‐4‐ol, myrcene, β‐ocimene, and limonene were reported to be the most abundantly present components in the fruits (Barrero et al., 2005; Delazar et al., 2004; Gourine et al., 2010; Mecherara‐Idjeri, Hassani, Castola, & Casanova, 2008) . The changes in major compounds in extracted essential oil from samples (amounts were >0.05) were presented in Table 2. No significant differences were observed between the five drying methods, nor compared to the fresh sample, regarding major components. The α‐pinene (46.045), α‐terpinen‐4‐ol (5.34), and limonene (8.835) compounds have the highest percentage in the volatile oil of fresh sample, while myrcene (15.285), β‐ocimene (4.2), and β‐caryophyllene (4.115) were highest values in shade drying. In a way, it can be said that drying in shadow condition resulted in preservation bioactive compounds. Drying of blueberry (*Vaccinium corymbosum* L.) fruits using hot air convective drying led to the reduction of total polyphenols content, content of anthocyanins and antioxidant capacity. However, combination of this method with microwave vacuum drying caused a significant increase in product quality also reduce drying time (Zielinska & Michalska, 2016).

**Table 2:** The effects of different drying methods (include sun, shade, oven, and microwave) on major compounds in the essential oil of the *Pistacia atlantica* seeds

Method	Quantity (%)	Major components					
		β‐Caryophyllene	α‐Terpine‐4‐ol	β‐Ocimene	Limonene	Myrcene	α‐Pinene
Fresh	0.216^a^	1.415^b^	5.34^a^	3.325^a^	8.835^a^	10.235^a^	46.045^a^
Sun	0.126^b^	3.4^ab^	4.015^a^	2.01^a^	0.05^b^	11.215^a^	0.126 ^b^
Microwave	0.09^c^	3.175^ab^	2.285^a^	2.665^a^	0.605^b^	13.025^a^	0.09^c^
Oven 40°C)	0.208^a^	2.1^b^	3.485^a^	2.175^a^	0.96^b^	9.185^a^	22.195^a^
Oven 60°C)	0.168^a^	2.97^ab^	2.095^a^	1.8^a^	1.095^b^	9.555^a^	0.168^ab^
Shade	0.105^c^	4.115^a^	5.81^a^	4.2^a^	1.55^b^	15.285^a^	0.105^c^

Note

In each row, different letters mean significant differences *p* < .05 according to the least significant difference test (LSD).

### 3.3 Effects of drying on *P. atlantica* texture

**Figure 3:**
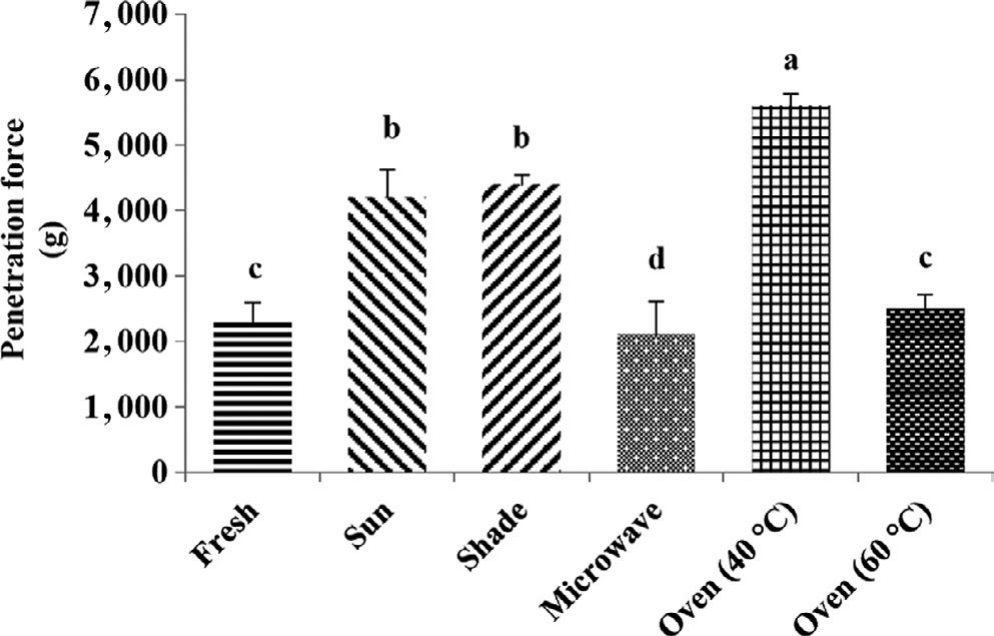
Penetration force means values for different drying methods

Texture is one of the most affected features, which is conditioned by the impact of drying conditions on food structure. The hardness is determined as the maximum force in the force–deformation curve; however, the brittleness is defined through the number of peaks (Aguilera et al., 2003). As can be seen in Figure 3, different drying methods significantly affected the shrinkage of *P. atlantica* seeds (*p* < .05). The hardness value of fresh sample was 2,300 g; the highest score for the penetration force was observed in oven 40°C (5,600 g) following shade (4,400 g), sun (4,200 g), oven 60°C (2,500 g), and microwave (2,100 g). The minimum value of penetration force was recorded in the microwave method, this may as a result of a more rapidly removing the moisture from the texture, so samples dried by microwave are more porous and brittle. In the 40°C oven, the drying rate is low, and by incrementing the temperature from 40 to 60°C, the texture becomes harder; the penetration force significantly decreases which is due to a slower removal of moisture during drying and wrinkling of the texture.

### 3.4 Modeling

Table 3 shows the drying data fitted to six different mathematical models. By comparing the *R* ^2^ and RMSE between the observed and predicted variables, the performance of these models was investigated. To better estimate the drying curves, the equations with highest *R* ^2^ (closer to 1) and lowest RMSE (closer to 0) were selected (Arumuganathan et al., 2009; Togrul & Pehlivan, 2003). The comparison of experimental data with those predicted with some models for *P. atlantica* samples is presented in Table 3, at different drying methods. It appears, from Table 3, that the most appropriate model is the Midilli–Kucuk model in 40°C oven (with *R* ^2^ = .9969 and RMSE = 0.02), 60°C oven (with *R* ^2^ = .9788 and RMSE = 0.053), sun (with *R* ^2^ = .9977 and RMSE = 0.02), and microwave drying (with *R* ^2^ = .9995 and RMSE = 0.008). The best suitable model is the Wang and Singh model in shade with *R* ^2^ = .9961 and RMSE = 0.019.

**Table 3:** Results of statistical analysis on the modeling of drying methods for *P. atlantica* seeds

Mathematical models		Drying methods				
		Oven (40°C)	Oven (60°C)	Sun	Microwave	Shade
Wang and Singh	a	−0.033	−0.082	−0.073	−0.079	−0.014
	b	−0.0003	0.002	0.001	0.002	0.0004
	*R* ^2^	.9968	.963	.8689	.9922	.9961
	RMSE	0.018	0.063	0.127	0.028	0.019
Logarithmic	a	111	1.11	0.937	1.07	–
	c	−110	−0.061	0.071	−0.021	–
	k	0.0003	0.095	.174	.095	–
	*R* ^2^	.9951	.9654	0.9918	0.9895	–
	RMSE	0.023	0.064	0.035	0.034	–
Henderson–Pabis	a	1.08	1.06	0.99	1.054	–
	k	0.075	0.107	0.137	0.099	–
	*R* ^2^	0.9576	0.9637	0.967	0.9892	–
	RMSE	0.074	0.062	0.064	0.032	–
Page	k	0.017	0.016	0.27	0.055	0.137
	*n*	1.58	2.27	0.69	1.25	1.046
	*R* ^2^	.9957	.9784	.9743	.9941	.9865
	RMSE	0.0212	0.048	0.056	0.024	0.035
Newton	k	0.063	0.093	0.137	0.0911	–
	*R* ^2^	.9507	.9536	.9673	.9826	–
	RMSE	0.087	0.067	0.059	0.039	–
Midilli–Kucuk	a	0.986	0.978	1	0.99	–
	b	−0.057	−0.285	0.0025	0.003	–
	k	−0.037	−0.256	0.079	0.039	–
	*n*	0.711	0.643	1.32	1.45	–
	*R* ^2^	.9969	.9788	.9977	.9995	–
	RMSE	0.02	0.053	0.02	0.008	–

Abbreviations: *R* ^2^, coefficient of determination; RMSE, root mean square error.

For drying of thin‐layer carrot slices using microwave oven, the Midilli–Kucuk is the most appropriate model (Zarein et al., 2013). In a study by Gharehbeglou et al. (2014), the modified Henderson and Pabis in 85°C was chosen to better estimate the drying curves regard to drying of turnip. In a study, Aktaş and Polat (Aktas & Polat, 2007) showed that to explain the drying behaviors of two famous cultivars of Turkish pistachios, Kirmizi and Siirt Henderson–Pabis and the modified Pabis models are the most appropriate models. In solar drying of Sultana grapes, a two‐term drying model could satisfactorily define the curve (Yaldiz, Ertekin, & Uzun, 2001).

## 4 CONCLUSION

Considering the prevailing insecurity in food supplies throughout the world, cost‐effective and hygienic ways of drying foods are crucial. This study tried to assess the performance of some seed drying procedures in terms of physiological quality of *P. atlantica* seeds. The results indicated that microwave drying has afforded higher moisture removal in a shorter period of time compared with other mentioned methods. Moreover, it was found that the essential oil composition was not considerably influenced by microwave method and the quality of the texture is appropriate. The results of this study are expected to help finding the best ways for drying of *P. atlantica* seeds and will constitute valuable data for industrial uses. Retention indices of various components in extracted essential oil have been compared to previous studies in Table 4 that indicated the results were in compliance with previous studies.

**Table 4:** Chemical composition of Pistacia Atlantica essential oil

Components	RI	RI (ref)
β‐Caryophyllene	1,418	1,412
α‐Terpine−4‐ol	1,046	1,056
β‐Ocimene	1,040	1,036
Limonene	1,031	1,028.24
Myrcene	991	991
α‐Pinene	939	939

Abbreviations: RI, Retention index calculation using a temperature program according to n‐alkanes. RI (ref), Retention index described by Sedaghat Boroujeni et al. tr, traces (%<.01).

## CONFLICT OF INTEREST

The authors declare that they do not have any conflict of interest against the publication the present study.

## ETHICAL APPROVAL

This study does not involve any human or animal testing.
